# Autophagy mediates tumor necrosis factor-α-induced phenotype switching in vascular smooth muscle A7r5 cell line

**DOI:** 10.1371/journal.pone.0197210

**Published:** 2018-05-11

**Authors:** Marina García-Miguel, Jaime A. Riquelme, Ignacio Norambuena-Soto, Pablo E. Morales, Fernanda Sanhueza-Olivares, Constanza Nuñez-Soto, David Mondaca-Ruff, Nicole Cancino-Arenas, Alejandra San Martín, Mario Chiong

**Affiliations:** 1 Advanced Center for Chronic Disease (ACCDiS), Center for studies of Exercise, Metabolism and Cancer (CEMC), Facultad de Ciencias Químicas y Farmacéuticas, Universidad de Chile, Santiago, Chile; 2 Division of Cardiology, Department of Medicine, Emory University, Atlanta, Georgia, United States of America; Qatar University College of Health Sciences, QATAR

## Abstract

Vascular smooth muscle cells (VSMC) dedifferentiation from a contractile to a synthetic phenotype contributes to atherosclerosis. Atherosclerotic tissue has a chronic inflammatory component with high levels of tumor necrosis factor-α (TNF-α). VSMC of atheromatous plaques have increased autophagy, a mechanism responsible for protein and intracellular organelle degradation. The aim of this study was to evaluate whether TNF-α induces phenotype switching of VSMCs and whether this effect depends on autophagy. Rat aortic Vascular smooth A7r5 cell line was used as a model to examine the phenotype switching and autophagy. These cells were stimulated with TNF-α 100 ng/mL. Autophagy was determined by measuring LC3-II and p62 protein levels. Autophagy was inhibited using chloroquine and siRNA Beclin1. Cell dedifferentiation was evaluated by measuring the expression of contractile proteins α-SMA and SM22, extracellular matrix protein osteopontin and type I collagen levels. Cell proliferation was measured by [^3^H]-thymidine incorporation and MTT assay, and migration was evaluated by wound healing and transwell assays. Expression of IL-1β, IL-6 and IL-10 was assessed by ELISA. TNF-α induced autophagy as determined by increased LC3-II (1.91±0.21, *p*<0.001) and decreased p62 (0.86±0.02, *p*<0.05) when compared to control. Additionally, TNF-α decreased α-SMA (0.74±0.12, p<0.05) and SM22 (0.54±0.01, *p*<0.01) protein levels. Consequently, TNF-α induced migration (1.25±0.05, *p*<0.05), proliferation (2.33±0.24, *p*<0.05), and the secretion of IL-6 (258±53, *p*<0.01), type I collagen (3.09±0.85, *p*<0.01) and osteopontin (2.32±0.46, *p*<0.01). Inhibition of autophagy prevented all the TNF-α-induced phenotypic changes. TNF-α induces phenotype switching in A7r5 cell line by a mechanism that required autophagy. Therefore, autophagy may be a potential therapeutic target for the treatment of atherosclerosis.

## Introduction

Vascular smooth muscle cells (VSMCs) synthetic phenotype is characterized by an increased migration, proliferation and extra-cellular matrix (ECM) secretion [1]. However, differentiated VSMC have reduced migration and proliferation as well as low synthesis of ECM components, but increased contractile protein content (contractile phenotype) [1]. Following vascular injury, contractile and differentiated VSMC undergo transient phenotypic modifications that promote their regression to a synthetic and dedifferentiated state that allows the vascular injury recovery [2]. However, the high plasticity of VSMC may also have harmful consequences, since abnormal phenotypic changes may contribute to the progression of vascular diseases, such as atherosclerosis and hypertension [3].

Vascular remodeling and chronic inflammation characterize Atherosclerosis. Indeed, the inflammatory cytokine TNF-α expression increases in atherosclerotic tissue and contributes to VSMC migration and proliferation [4] by a mechanism that is not fully understood. On the other hand, increased autophagy has been described in VSMC within the atherosclerotic plaque [5]. Autophagy, one of the main form of protein degradation, is a tightly regulated process that begins with the formation of a double membrane vesicle called the autophagosome that fuses with the lysosome to promote the degradation and recycling of cytoplasmic content [6, 7]. Even though autophagy has been described to be important in the degradation of the contractile machinery [8], its role in the TNF-α-induced VSMC phenotypic change remains to be elucidated.

In this work, we used A7r5 cell line as a VSMC model to examine the phenotype switching and autophagy. We showed that TNF-α induces autophagy in A7r5 cells by a NF-kB-dependent mechanism. More importantly, TNF-α induces dedifferentiation, along with a migratory, proliferative and inflammatory phenotype in A7r5 cells through an autophagy-dependent mechanism. Altogether, our findings suggest that autophagy is a key mediator on the TNF-α-induced phenotype switching in A7r5 cells.

## Material and methods

### Cell culture

The smooth muscle A7r5 cell line was obtained from the thoracic aorta of BDIX rat (*Rattus novergicus)* [9]. The A7r5 cells were purchased from ATCC (CRL-1444) and were expanded in DMEM supplemented with 2 mM glutamine and 10% FBS as described [10, 11]. Prior to experiments, culture media was replaced with DMEM 2 mM glutamine and 2% FBS and cells were cultured for further 24 h. Total serum deprivation induced autophagy (S1 Fig). In that condition, effects of TNF-α on autophagy could not be assessed. Cells were maintained in a standard incubator at 37 °C with 95% O_2_ and 5% CO_2_ and used for experiments at 80–90% confluency.

### Western blot analysis

Cell cultures were lysed with RIPA lysis buffer (Tris-HCl 10 mmol/L, EDTA 5 mmol/L, NaCl 50 mmol/L, 1% deoxycholic acid and 1% triton X-100, pH 7.4). Protein concentration was determined by the Bradford method (BioRad protein assay). Equal amounts of proteins from cell extracts were separated by SDS-PAGE 8–15%, electro-transferred to PVDF membranes and blocked with 5% milk. Primary antibodies were used against type I collagen 1:2000 (cat #ABT123 Millipore), p62 1:2000 (cat #5114 Cell Signaling), osteopontin 1:2000 (cat #ab8448 Abcam), Beclin1 1:2000 (cat #3738 Cell Signaling), α-SMA 1:20000 (cat #ab7817 Abcam), GAPDH 1:50000 (cat #8795 Sigma), SM22 1:10000 (cat #ab14106 Abcam), LC3-I/II 1:1000 (cat #2775 Cell Signaling). Membranes were re-blotted with a horseradish peroxidase-linked secondary antibody 1:5000 (mouse cat #402335 and rabbit cat#401315 Merck). Bands were detected using ECL (Biological Industries) and luminescence was assessed using a digital imaging system (Syngene). Quantification of the bands by densitometry was performed using UN-SCAN-IT gel software.

### Quantification of autophagy

To evaluate autophagic flux, A7r5 cells were treated with chloroquine (CQ) 20 μmol/L during the last 4 h of the 24 h stimulus with TNF-α 100 ng/mL. Also, treatment with CQ 5 μmol/L for 24 h was used to evaluate the effects of autophagy on the expression of contractile and ECM proteins. To genetically inhibit autophagy, cells were transfected with 100 nmol/L scrambled siRNA or siRNA against Beclin1 (Sigma) using oligofectamine (Life Technologies) in Optimem medium (Life Technologies) during 24 h, following the manufacturer’s instructions.

To visualize autophagosomic vesicles, A7r5 cells were seeded in 12 well plates with 18 mm glass coverslips containing 2 x 10^5^ cells per well. Cells were maintained with 2% FBS for 24 h and transduced with an adenovirus Ad LC3-GFP for 24 h using a multiplicity of infection (MOI) of 150. After transduction, cells were stimulated with TNF-α 100 ng/mL for 24 h in the presence or absence of CQ. Then, cells were washed with PBS and fixed with 4% paraformaldehyde. The coverslips were treated with Hoechst (1:1000) to stain the nuclei and images were analyzed using a Carl Zeiss Pascal 5 confocal microscopy.

### Immunofluorescence

Actin organization was visualized in A7r5 cells fixed with 4% paraformaldehyde for 20 min and permeabilized with 0.1% Triton X-100 for 25 min. After blocking with 3% BSA, cells were stained with rhodamine-phalloidin (1:1500). Hoechst (1:000) was used to stain the nuclei. Cells were visualized with an epifluorescence microscope (Leica DM IL LED Fluo).

### Migration and proliferation assays

Proliferation of VSMC was determined using MTT assay [12] and [^3^H]-thymidine incorporation [11]. A7r5 migration was evaluated by the wound healing and transwell assays in the presence of bromodeoxiuridine to inhibit cell proliferation [11].

### Zymography

Electrophoresis was performed with the supernatant of cell cultures using 7.5% polyacrylamide gels with gelatin 8.5 mg/mL. Proteins in the gel were renatured by using 2.5% Triton-X-100 in PBS for 30 min, incubated with Tris-HCl 50 mmol/L pH 8, NaCl 200 mmol/L, CaCl_2_ 1.25 mmol/L for 18 h at 37°C and then stained with Coomassie blue (Merck).

### Interleukin determination

The levels of IL-1β, IL-6 and IL-10 were measured from A7r5 cell supernatants using Quantikine ELISA kits (R&D Systems), according to the manufacturer’s instructions. For quantitative RT-PCR, total RNA was extracted with TRIzol reagent (Invitrogen) and reverse transcribed. Equal amounts of cDNA were subjected to real-time PCR using SYBR green (Applied Biosystems) as described [13]. Data were normalized using Pabpn1 as endogenous control using the Pfaffl method [14]. Primers were: IL-6 rat forward 5’-ACT GCC TTC CCT ACT TCA CAA GTC-3’ & reverse 5’-ACT CCA GGT AGA AAC GGA ACT CCA-3’; Pabpn1 rat forward 5’- GTT GGC AAT GTG GAC TAT GG-3’ & reverse 5’-AAC AGG GAC TCA TCT AAG GC-3’.

### Statistical analysis

Results are shown as mean ± SEM of at least three independent experiments. Data was analyzed by *t* test or ANOVA, depending on the conditions. Post-tests are indicated in each figure legend. Differences were considered significant at *p*<0.05.

## Results

### TNF-α induces autophagy through an IKKα-dependent mechanism in A7r5 cells

**Fig 1:**
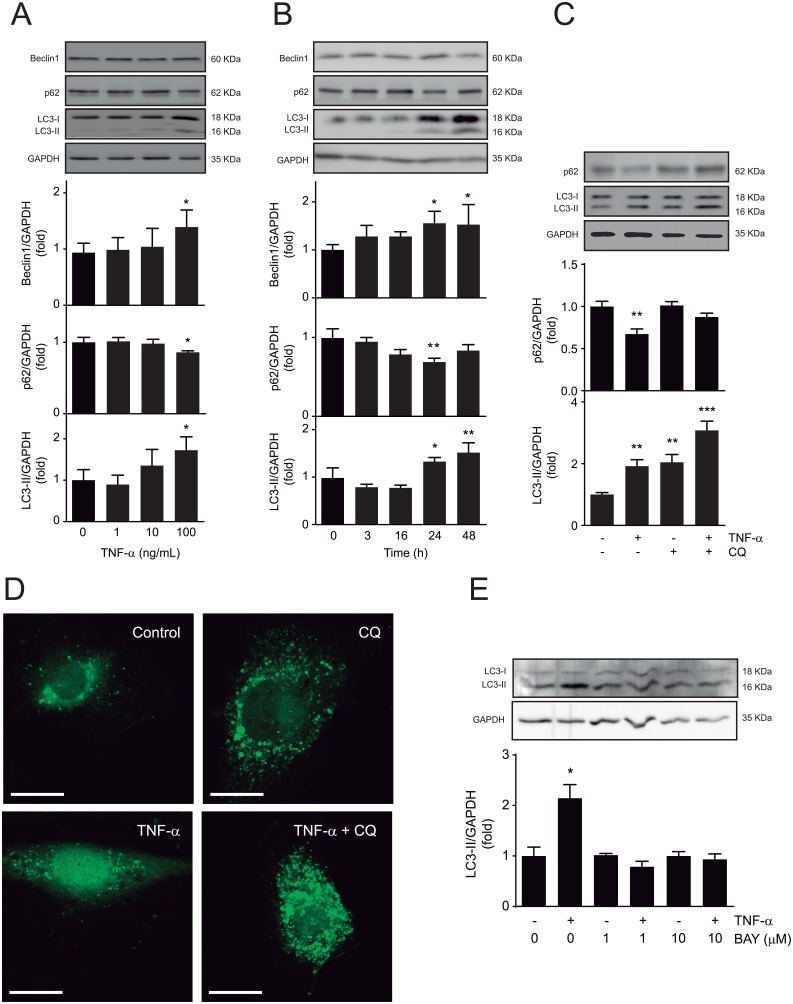
TNF-α activates autophagy in A7r5 cells through an-IKKα dependent pathway. (**A**) Western blot analysis of Beclin1, p62, LC3-II and GAPDH in A7r5 cells stimulated with 0, 1, 10 and 100 ng/mL of TNF-α (n = 4; *p<0.05 vs 0 ng/mL TNF-α). (**B**) Western blot analysis of Beclin1, p62, LC3-II and GAPDH in A7r5 cells incubated with TNF-α (100 ng/mL) at 0, 3, 16, 24 and 48 h (n = 4; *p<0.05, **p<0.01 vs 0 h). (**C**) Western blot analysis of p62, LC3-II and GAPDH in A7r5 cells treated with TNF-α (100 ng/mL) for 24 h and co-administered with or without chloroquine (CQ, 20 μmol/L) during the last 4 h of stimulus (n = 3; **p<0.01, ***p<0.001 vs control. (**D**) Visualization of autophagic vesicles by confocal microscopy in A7r5 cells transduced with adenovirus overexpressing LC3-GFP at MOI = 150 for 24 h and incubated with TNF-α (100 ng/mL) for 24 h and co-administered with or without CQ (20 μmol/L) during the last 4 h of TNF-α stimulus (n = 3). (**E**) Western blot analysis of LC3-II in A7r5 cells pre-treated with or without BAY-11-7082 (1 and 10 μmol/L) for 30 min, followed by incubation with TNF-α (100 ng/mL) for 24 h (n = 3; *p<0.05 vs control). Data are expressed as mean ± SEM and analyzed by one-way ANOVA followed Dunnett post-test (**A**, **B** and **C**) and paired *t* test comparing each condition to its control (**E**).

A7r5 cells were stimulated with TNF-α 0, 1, 10 and 100 ng/mL for 24 h and autophagy markers p62, Beclin1 and LC3-II were determined by western blot. The results showed that TNF-α 100 ng/mL induced p62 decrease (0.86±0.02, *p*<0.05) and LC3-II increase (2.45±0.45, *p*<0.05) (Fig 1A). Additionally, treatment of A7r5 cells with TNF-α (100 ng/mL) at 0, 3, 16, 24 and 48 h showed an increase in Beclin1 and LC3-II at 24 h (1.55±0.24, *p*<0.05 and 1.81±0.24, *p*<0.01, respectively) (Fig 1B). These results suggest that TNF-α activates autophagy. TNF-α effect on autophagy is not specific because serum deprivation and platelet derived growth factor-BB (PDGF-BB) also induced LC3-II increase (S1 Fig). In order to discriminate if the increase in LC3-II levels was the result of augmented autophagosome formation or decreased degradation of the autophagosomes by lysosomes, we performed these measurement in the presence of chloroquine (CQ) [15], which inhibits the fusion of the autophagosomes with the lysosome [16]. The results showed that the addition of CQ during the last 4 h of the 24 h treatment with TNF-α (100 ng/mL) further increased LC3-II levels (2.13±0.25, *p*<0.01), whereas p62 was restored to baseline (Fig 1C), suggesting that TNF-α induces autophagic flux. To confirm these findings, A7r5 cells were transduced with an adenovirus containing LC3 bound to GFP, to visualize autophagic vesicles. Treatment of A7r5 cells with TNF-α for 24 h induced an increase in the number of LC3-positive autophagic puncta. Moreover, co-administration of TNF-α with CQ further increased the autophagic vesicles (Fig 1D). These data further support the interpretation that TNF-α increases autophagic flux.

It has been previously established that the NF-κB pathway is required for the induction of autophagy by multiple stimulus [17]. Therefore, considering that TNF-α activates NF-κB [18], we sought to evaluate if TNF-α-induced autophagy relies on NF-κB pathway activation. To test this, A7r5 cells were pre-treated for 30 min with BAY-117082, which irreversibly inhibits TNF-α-induced IKKα activation [19]. Results showed that pretreatment with BAY-117082 inhibited TNF-α-induced LC3-II (Fig 1E). Taken together, these results suggest that TNF-α activates autophagy in VSMC through an IKKα-dependent mechanism.

### Autophagy mediates dedifferentiation of A7r5 cells induced by TNF-α

**Fig 2:**
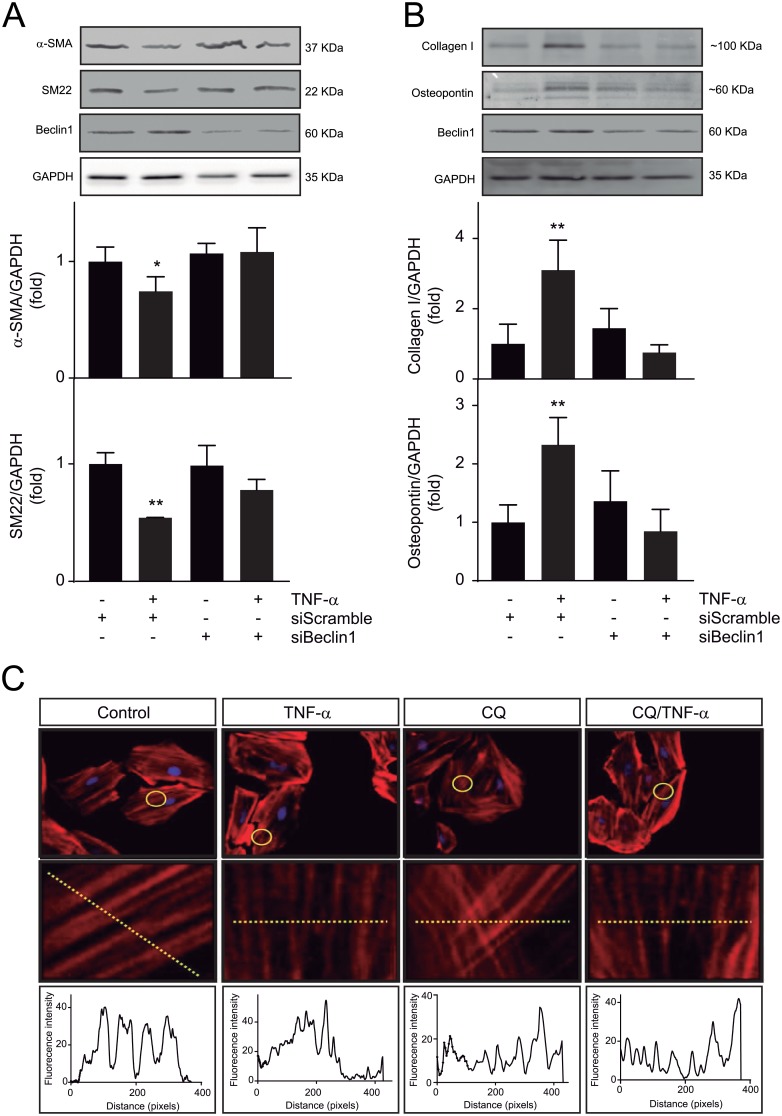
TNF-α induces dedifferentiation of A7r5 cells by an autophagy-dependent pathway. (**A**) Western blot analysis of α-SMA and SM22 (n = 3–4; *p<0.05, **p<0.01 vs control) or (**B**) collagen type I and osteopontin (n = 3–4; **p<0.01 vs control) in A7r5 cells incubated with TNF-α (100 ng/mL) for 48 h in the presence or absence of siScramble and siBeclin1. GAPDH was used as loading control. (**C**) Visualization of actin filaments in A7r5 cells stained with rhodamine-phalloidin after treatment with TNF-α (100 ng/mL) for 48 h in the presence or absence of chloroquine (CQ, 5 μmol/L) during the last 24 h of TNF-α stimulus. Lower panel represent a fluorescence intensity profile of the lines depicted on the images. Data are expressed as mean ± SEM and analyzed by two-way ANOVA, followed by Holm Sidak post-test.

To evaluate the role of TNF-α in A7r5 cell dedifferentiation, extracellular matrix (type I collagen and osteopontin) and contractile (α-SMA and SM22) protein levels were evaluated. A7r5 cells were stimulated with TNF-α (100 ng/mL) for 48 h and the aforementioned protein levels were determined by western blot. TNF-α induced an increase in type I collagen and osteopontin (3.09±0.85, *p*<0.01 and 2.32±0.46, *p*<0.01, respectively) (Fig 2A) and a reduction in α-SMA and SM22 levels (0.74±0.12, *p*<0.05 and 0.54±0.01, *p*<0.01, respectively) (Fig 2B). All TNF-α effects on these proteins was blocked by autophagy inhibition using siRNA against Beclin 1 (Fig 2A and 2B). Treatment with siRNA reduced 62±8% Beclin 1 protein levels (Fig 2A and 2B). Moreover, TNF-α (100 ng/mL) for 48 h also induced a disorganized pattern of the actin filaments of A7r5 cells stained with rhodamine-phalloidin. This effect was also abolished upon co-administration of CQ during the last 24 h of TNF-α stimulus (Fig 2C). Taken together, these results suggest that TNF-α induces A7r5 cell dedifferentiation through an autophagy-dependent mechanism.

### TNF-α requires autophagy to induce A7r5 cell migration

**Fig 3:**
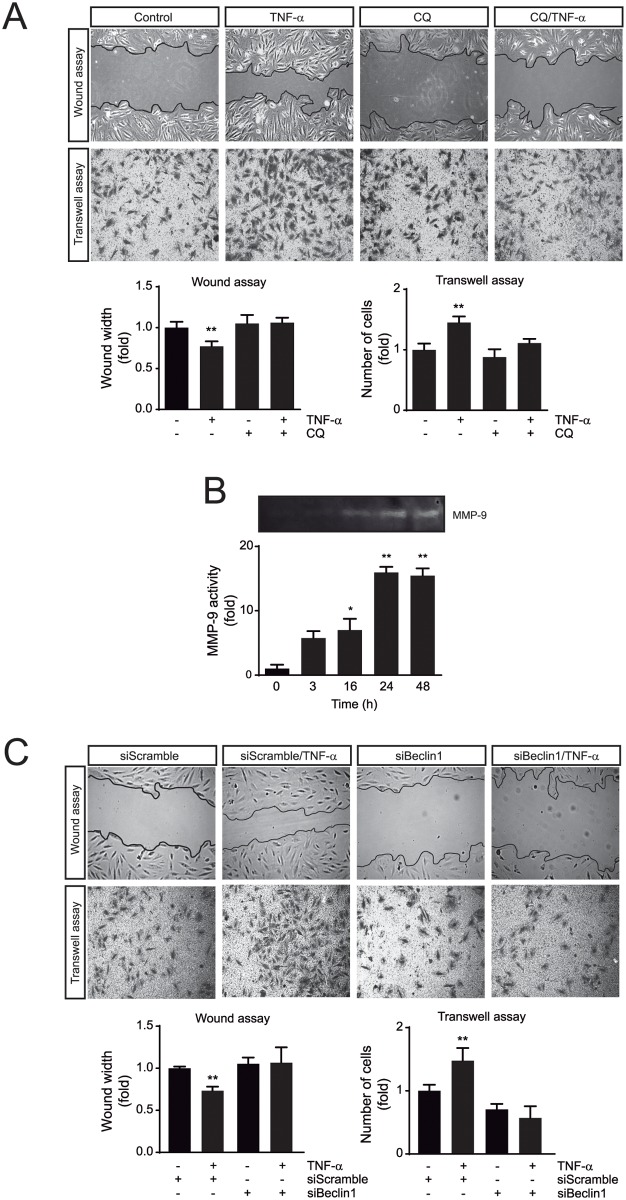
TNF-α requires autophagy to induce migration in A7r5 cells. (**A**) Assessment of migration by the wound healing and transwell assays in A7r5 cells stimulated with TNF-α (100 ng/mL) for 24 h in the presence or absence of chloroquine (CQ, 20 μmol/L) during the last 4 h of TNF-α stimulus (n = 4; **p<0.01 vs control) or (**C**) siScramble and siBeclin1 for 24 h (n = 4; **p<0.01 vs control). Migration was visualized using a phase contrast microscope (upper panels of **A** and **C**). The results of the wound healing and transwell assays were quantified by measuring wound width and the number of cells that migrated through the Boyden chamber, respectively (lower panels of **A** and **C**). (**B**) Zymography analysis of matrix metalloproteinase 9 (MMP-9) in A7r5 cells stimulated with TNF-α (100 ng/mL) for 24 h (n = 3; *p<0.05 vs control). Data are expressed as mean ± SEM and analyzed by one-way ANOVA, followed by Holm Sidak (**A** and **C**) and Dunnett (**B**) post-tests.

To test the role of autophagy in the TNF-α-induced migratory phenotype, A7r5 cells were subjected to a wound-healing and transwell assays in the presence of TNF-α (100 ng/mL) for 24 h in the presence or absence of CQ. The results showed that TNF-α induces migration measured by wound closure (0.73±0.05 fold vs control, *p*<0.01) and transmigration (1.25±0.09 fold vs control, *p*<0.05) (Fig 3A). The effect of TNF-α was blunted by autophagy inhibition with CQ (Fig 3A). Moreover, TNF-α also induced an increase of MMP-9 activity after 16 h of incubation (Fig 3B).

In order to confirm these results, autophagy initiation was also blocked by downregulation of Beclin1 using siRNA and TNF-α-induced migration and transmigration were evaluated. As presented in Fig 3C, autophagy inhibition by Beclin1 knock down also prevented TNF-α-induced migration and transmigration. Thus, our results showed that TNF-α induced A7r5 cell migration through an autophagy-dependent mechanism.

### TNF-α requires autophagy to induce A7r5 cell proliferation

**Fig 4:**
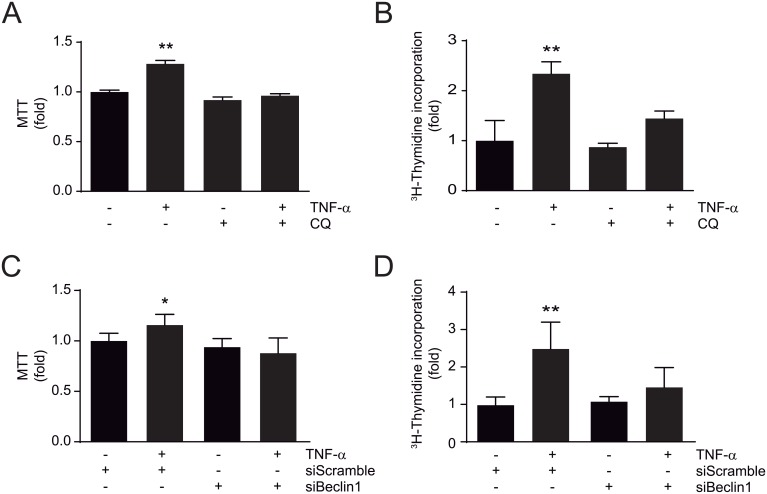
TNF-α induces proliferation of A7r5 cells through an autophagy-dependent mechanism. (**A**) Determination of proliferation by the MTT assay and (**B**) incorporation of [^3^H]-thymidine in A7r5 cells treated with TNF-α (100 ng/mL) for 24 h in the presence or absence of chloroquine (CQ, 20 μmol/L) during the last 4 h of TNF-α stimulus (n = 4; **p<0.01 vs control) or (**C** and **D**) siScramble and siBeclin1 (n = 4; *p<0.05, **p<0.01 vs control). Data are expressed as mean ± SEM and analyzed by one-way ANOVA, followed by Holm Sidak (**A** and **C**) and Dunnett (**B** and **D**) post-tests.

To study whether autophagy is necessary for TNF-α-induced A7r5 cell proliferation, A7r5 cells were treated with TNF-α (100 ng/mL) for 24 h in the presence or absence of autophagy inhibition. TNF-α induced A7r5 cell proliferation assessed by the MTT assay and [^3^H]-thymidine incorporation (1.28±0.03, *p*<0.01 and 2.33±0.24, *p*<0.05, respectively) (Fig 4A and 4B). However, this effect was prevented by the addition of CQ. Furthermore, siRNA Beclin1 also abolished the TNF-α-induced A7r5 proliferation also measured by both MTT and [^3^H]-thymidine incorporation (1.15±0.1, *p*<0.01 and 2.48±0.71, *p*<0.01, respectively) (Fig 4C and 4D). Therefore, these findings suggest that TNF-α induces A7r5 cell proliferation through an autophagy dependent mechanism.

### TNF-α stimulates the release of interleukin (IL)-6 from A7r5 cells in an autophagy dependent manner

Currently, much remains to be elucidated in regards to cytokine expression and release in VSMC. Therefore, we sought to evaluate if these cells develop an inflammatory phenotype upon TNF-α administration and whether it depends on autophagy. A7r5 cells do not express detectable baseline or TNF-α-induced levels of IL-1β and IL-10 measured by ELISA and RT-qPCR (data not shown).

**Fig 5:**
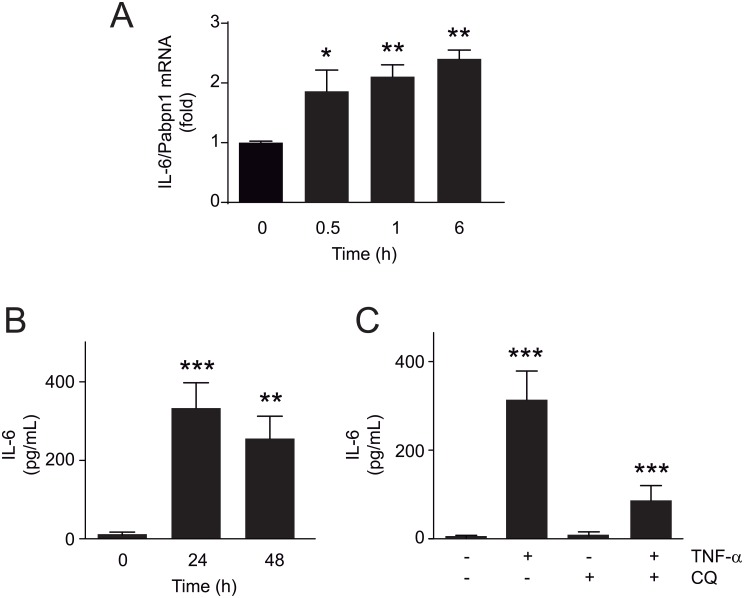
Autophagy mediates TNF-α-induced secretion of IL-6 in A7r5 cells. (**A**) Determination of IL-6 mRNA using RT-qPCR in A7r5 cells treated with TNF-α (100 ng/mL) for 30 min, 1 and 6 h (n = 6; *p<0.05, **p<0.01 vs 0 h). (**B**) Determination of IL-6 using ELISA assay in A7r5 cells treated with TNF-α (100 ng/mL) for 24 and 48 h (n = 4; **p<0.01, ***p<0.001 vs 0 h) or (**C**) co-administered with or without chloroquine (CQ, 20 μmol/L) during the last 4 h of the 24 h stimulus with TNF-α (n = 4; ***p<0.001 vs control). Data are expressed as mean ± SEM and analyzed by one-way ANOVA, followed by Dunnett and Tukey post-tests.

IL-6 is a pro-inflammatory cytokine that is also relevant to the atherosclerotic process and is known to be expressed in VSMC [20]. Fig 5A shows that stimulation of A7r5 cells with TNF-α (100 ng/mL) for 30 min, 1 and 6 h increased IL-6 mRNA levels (1.86±0.33, p<0.05; 2.11±0.18, p<0.01; 2.41±0.13, p<0.01, respectively). TNF-α (100 ng/mL) for 24 and 48 h also increased IL-6 release at both times (334±65, *p*<0.01 and 258±53, *p*<0.01, respectively) (Fig 5B). Moreover, inhibition of autophagy with CQ during the last 4 h of TNF-α stimulus, totally prevent TNF-α-induced IL-6 release (Fig 5C). Thus, results show that TNF-α induces IL-6 release in A7r5 cells through an autophagy-dependent mechanism.

## Discussion

TNF-α is one of the most relevant cytokines present in the blood and tissues of atherosclerotic patients [21, 22]. This work shows that TNF-α induces vascular smooth muscle A7r5 cell dedifferentiation, as determined by a reduction in contractile proteins, an increase in metalloprotease and extracellular matrix proteins, and induction of migratory and proliferative phenotypes. Moreover, TNF-α induces IL-6 but not IL-1β or IL-10 release in A7r5 cells. We also showed that TNF-α-induced A7r5 cell dedifferentiation occurs through an autophagy dependent mechanism.

It has been previously described by Jia *et al*. that TNF-α induces autophagy in VSMC [23]. However, they only evaluated the autophagic markers Beclin1 and LC3-II without assessing autophagic flux [23]. Our results show that TNF-α induces autophagy in vascular smooth muscle A7r5 cell by measuring Beclin1, p62, LC3-II and autophagosome contents. By inhibiting autophagosome-lysosome fusion with CQ, we also found that TNF-α activates autophagy flux. Our results also showed that TNF-α-dependent autophagy induction in VSMC occurs through an IKKα-dependent mechanism. Criollo *et al*. demonstrated that classical autophagy inductors, such as starvation and rapamycin, requires IKK complex for optimal induction of autophagy *in vivo* [17]. Moreover, they demonstrated that IKK activation triggers autophagy which is not correlated with NF-kB activation [17].

It has been previously demonstrated that VSMC undergoing vascular remodeling show a higher proliferation and migration rate and TNF-α also promote these features [24, 25]. However, the role of autophagy in these effects was unknown. Here we described that TNF-α-induced vascular smooth muscle A7r5 cell dedifferentiation, as well as migration and proliferation, were prevented by autophagy inhibition. These data suggest that autophagy is an important phenomena for VSMC phenotype switching.

Autophagy was recently reported to be necessary for the motility of metastatic cancer cells [26]. In these cells, autophagy promotes focal adhesion turnover through a mechanism that involves the degradation of paxillin, which is a key focal adhesion protein. Furthermore, paxillin interacts with LC3 through a LIR motif that is regulated by Src [26]. On the other hand, autophagy may participate in the induction of proliferation of VSMC in response to various stimuli, including TNF-α. Treatment of these cells with Sonic hedgehog protein (Shh) induces autophagy and promotes proliferation in an Akt dependent manner [27]. Moreover, autophagy induced by PDGF-BB is also associated with increased VSMC proliferation and migration and its inhibition using 3-methyladenine or spautin-1, reduces these effects [28]. Furthermore, defective autophagic activity in VSMC induces G1-cell cycle arrest, increases migration and up-regulates MMP-9, transforming growth factor β (TGF- β), chemokine (C-X-C) motif ligand 12 (CXCL12) and elicits post-injury neointima formation [29].

TNF-α is a major pro-inflammatory mediator in several cell types, especially in the immune system [18]. We observed that although TNF-α induced a robust autophagy activation and A7r5 cell phenotype switching, its effect on cytokine release was minor. Indeed, our results show that TNF-α increased IL-6 release but not IL-1β and IL-10. This finding appears to be a specific feature of VSMC, given that TNF-α strongly increases cytokine secretion in other cell models [18, 30]. This may be due to the absence of these cytokines in rat VSMC. Nonetheless, the expression of both cytokines has been described in VSMC from primates, along with an increase in IL-1β and a reduction of IL-10 in response to aging [31].

Alternatively, Dupont *et al*. reported that autophagy mediates an unconventional secretory pathway of IL-1β release [32]. This secretory pathway depends on the Atg5, inflammasome, and one of the two mammalian Golgi reassembly stacking protein (GRASP) paralogues GRASP55 and Rab8a [32]. Furthermore, it has been shown that the autophagy-dependent secretion of IL-1β by the AIM2 inflammasome requires the microtubule associated protein EB1 in nasopharyngeal carcinoma and monocyte cell lines [33]. Also, the dectin-1/Syk pathway induces unconventional vesicle-mediated protein secretion that depends on both the inflammasome and autophagy in human macrophages [34]. Thus, future studies should evaluate whether TNF-α triggered IL-6 secretion in VSMC is achieved through similar mechanisms described for cancer cells and macrophages.

Regarding autophagy, there are pharmacological compounds that specifically regulate autophagic activity, such as CQ and spautin-1. In this context, it has been described that spautin-1 inhibits autophagy in an Akt-independent pathway inhibiting PDGF-induced hyperproliferation of VSMC [28]. Accordingly, our findings show a similar effect with CQ, which prevented the migratory and proliferative phenotype of VSMC. Further, the use of CQ in humans is currently approved and has showed beneficial effects in reducing the progression of pulmonary hypertension in murine models [35]. The current challenge is to find an autophagy modulator that can specifically discriminate between basal and maladaptive autophagy, which would allow targeting only the defective metabolic pathways of the disease.

In conclusion, our findings suggest that TNF-α elicits a VSMC phenotype switching through an autophagy-dependent mechanism.

## Supporting information

S1 Fig Serum deprivation and PDGF-BB activates autophagy in A7r5 cells. **(A)** Western blot analysis of LC3-II and GAPDH in A7r5 cells cultured during 24 h in DMEM + 2 mM pyruvate supplemented with 0, 2, 5 and 10% FBS (n = 3; *p<0.05 vs 10% FBS). (B) Western blot analysis of LC3-II and GAPDH in A7r5 cells incubated with PDGF-BB (10 nM) at 0, 6, 24 and 48 h (n = 4; *p<0.05 vs 0 h). Data are expressed as mean ± SEM and analyzed by one-way ANOVA followed Dunnett post-test.
(EPS)
